# How Can Peer Group Influence the Behavior of Adolescents: Explanatory Model

**DOI:** 10.5539/gjhs.v4n2p26

**Published:** 2012-03-01

**Authors:** Gina Tomé, Margarida Gaspar de Matos, Celeste Simões, Inês Camacho, José AlvesDiniz

**Affiliations:** T. University of Lisbon, Portugal

**Keywords:** Risk behaviour, Violence, Health, Well-being, Peer relation, Parent relation

## Abstract

The current work aims to study both the peer group and family influence on adolescent behaviour. In order to achieve the aforementioned objective, an explanatory model based on the Structural Equations Modelling (SEM)was proposed. The sample used was the group of adolescents that participated in the Portuguese survey of the European study Health Behaviour in School-aged Children (HBSC). The Portuguese survey included students from grades 6, 8 and 10 within the public education system, with an average age of 14 years old (SD=1.89). The total sample of the HBSC study carried out in 2006 was 4,877; however with the use of the SEM, 1,238 participants were lost out of the total sample.

The results show that peers have a direct influence in adolescents’ risk behaviours. The relationship with parents did not demonstrate the expected mediation effect, with the exception of the following elements: relation between type of friends and risk behaviour; and communication with parent and lesser involvement in violence behaviours and increased well-being. The negative influence of the peer group is more connected to the involvement in risk behaviours, whilst the positive influence is more connected with protective behaviours.

## 1. Introduction

There are a variety of negative health indicators that show a peek during adolescence, namely homicide rates, non-intentional injuries, driving under alcohol effect or infection by sexually-transmitted diseases (Mulye, Park, Neson, Irwin & Brindis, 2009). Experimenting substances also occurs usually during adolescents, a time of development in which tolerance is lower and the risk of dependency increases (Glaser, Shelton & Bree, 2010). Peers and family have a key role in promoting health during adolescence, as well as, the perception that youngsters have of their quality of life and subjective well-being. Health does not depend solely on the delivery of health care during illness; on the contrary, influence of different settings may be crucial (Gaspar & Matos, 2008).

Behavioural problems that occur during infancy and adolescence (particularly external problems, such as substance use and violence behaviours) may continue throughout adulthood, associated to social non-adaptation, substance abuse and conflicts (Bongers, Koot, Van der Ende & Verhulst, 2008). The peer group may on one hand, serve as a model and influence behaviours and attitudes, whilst on the other hand, it may provide easy access, encouragement and an appropriate social setting for consumption (Glaser, Shelton & Bree, 2010). Social Learning Theory suggests that it is not necessary for adolescents to observe a given behaviour and adopt it; it is sufficient to perceive that the peer group accepts it, in order to be able to opt for similar behaviours (Petraitis, Flay & Miller, 1995).

Peers may strongly determine preference in the way of dressing, speaking, using illicit substances, sexual behaviour, adopting and accepting violence, adopting criminal and anti-social behaviours and in many other areas of the adolescent’s life (Padilla, Walker & Bean, 2009; Tomé, Matos & Diniz, 2008). An example of this is that the main motives for alcohol consumption given by adolescents are related to social events, which usually take place in the company of friends, namely: *drinking makes holidays more fun, it facilitates approaching others, it helps relaxing or facilitates sharing experiences and feelings* (Kuntsche, Knibbe, Gmel & Engels, 2005). Also, mimicking risk behaviours may be greater when consumption begins in the context of a social event (Larsen, Engels, Souren, Granic & Overbeek, 2010).

On the other hand, having friends allows to share experiences and feelings and to learn how to solve conflicts. Not having friends, on the other hand, leads to social isolation and limited social contacts, as there are fewer opportunities to develop new relations and social interactional skills. Friendship is also positively associated to psychological well-being (Ueno, 2004), whilst a conflicting relation with peers is negatively associated with health (Laftman & Östberg, 2006). Stronger friendships may provide adolescents with an appropriate environment to development in a healthy way and to achieve good academic results. Adolescents with reciprocal friendships mention high levels of feelings of belonging in school; at the same time, reciprocity and feelings of belonging have positive effects in academic results (Vaquera & Kao, 2008).

School is a setting where interpersonal relations are promoted, which are important for youngsters’ personal and social development (Ruini *et al.*, 2009); it is responsible for the transmission of behavioural norms and standards and it represents an essential role in the adolescent’s socialisation process. The school is able to gather different peer communities and to promote self-esteem and a harmonious development between adolescents, which makes it a privileged space for meetings and interactions (Baptista, Tomé, Matos, Gaspar & Cruz, 2008). Adolescents spend a great part of their time at school, which also makes it a privileged context for involvement in or protection from risk behaviours (Piko & Kovács, 2010). Camacho, Tomé, Matos, Gamito and Diniz (2010) confirmed that adolescents who like school were those that more often were part of a peer group without involvement in risk behaviours; whilst those that mentioned they did not have any friends reported that they liked school less.

Despite the positive influence of the peer group during adolescence, the higher the adolescent’s autonomy from the peer group, the higher his/her resilience against its influence. This resilience seems to increase with age, which may mean that it is associated with youngsters’ maturity; and girls emerge in several studies as more resilient than boys (Sumter, Bokhorst, Steinberg & Westenberg, 2009). Another factor that may be found in the influence of the peer group is the type of friendship, which adolescents maintain with their peer group: if friends are close they have a greater influence on the other’s behaviours (Glaser, Shelton & Bree, 2010). When the friendship is perceived as reciprocal and of quality, is exerts greater influence (Mercken, Snijders, Steglich, Vartiainen & Vries, 2010). Another factor, which has been identified as a possible factor of decreasing peer influence is assertive refusal. Adolescents that are able to maintain an assertive refusal are less susceptible to the group’s influence (Glaser, Shelton & Bree, 2010). These are only some variables identified as possible factors decreasing peer influence.

The relation with parents may be a mitigating factor of the negative influence by peers. Communicating family rules and parental style have been inversely associated to substance, alcohol and tobacco consumption during adolescence. This influence is essential for adolescents’ development up to adulthood. Communication between parents and adolescents emerges as a protective factor for alcohol, tobacco and substance use (Newman, Harrison & Dashiff, 2008).

Sen (2010) observed that family meals could lead to creating a closer relation between parents and adolescents, by strengthening a positive relationship and avoiding certain risk behaviours, such as substance use amongst girls and alcohol consumption, physical violence and robberies, amongst boys. These differences between genders may be due to a greater importance that girls attribute to family activities, but they do not reveal that boys are indifferent to them, only that the relation between genders may differ. Huebner and Howell (2003) verified that parental monitoring and communication with parents protected adolescents of both genders from being involved in risk behaviours.

Parental monitoring can be defined as parents’ knowledge about their children’s activities, who they hang out with and what they do. It has been associated to protection of various risk behaviours throughout adolescence, such as substance use or sexual behaviours. It may vary according to age, gender or ethnicity and it generally decreases with age (Westling, Andrews, Hampson & Peterson, 2008).

Tobler and Komro (2010) confirmed with a sample made up of 2,621 adolescents in the 6^th^ and 8^th^ grades that the role of parents in the prevention of substance use during adolescence is essential; and that communication and parental monitoring were the factors, which most contributed to those results.

The greater the parental monitorings, the lower the adolescents’ involvement in risk behaviour. Li, Stanton and Feigelman (2000) confirmed in a longitudinal study with afro-American children and adolescents, that parental monitoring was a very important factor in reducing risk behaviour. Parental monitoring emerged as inversely correlated with risk behaviours. The correlation persisted throughout age, suggesting that its protective effect is persistent in the long-term. Other studies found associations between parental monitoring and substance use and other behavioural problems amongst youth (Simons-Morton & Chen, 2005).

Rai and collaborators (2003) also found a positive influence associated with parental monitoring, namely protection against substance use and sexual behaviours, but not condom use. On the other hand, the peer group was found to influence all risk behaviours assessed by the authors. The youngsters that had the perception of the involvement of peers in certain behaviours were more involved in similar behaviours; the same was found for those that presented a problematic relationship with their parents.

Literature seems to suggest that the peer group has an important role throughout adolescence; nevertheless it may influence negatively adolescents’ risk behaviours, by enhancing their involvement in such actions. On the other hand, parents have a protective role in the same behaviours, generally associated with good communication and parental monitoring.

Taking into account the aforementioned findings, the aim of this study will be to analyse how peer influence is associated with: 1) risk behaviour, 2) violence, 3) health behaviour, 4) well-being and 5) feelings about school; and whether that influence may be moderated by adolescents’ relations with parents.

## 2. Method

### 2.1 Sample

The sample used was the group of adolescents that participated in the 2006 Portuguese survey (continental Portugal) of the European study Health Behaviour in School-aged Children (HBSC) (http://www.hbsc.org).

The HBSC Study started in 1982 with a team of researchers from Finland, Norway and England and from 1985/86 it has been carried out every four years. Throughout the years, the study has grown in importance and currently there are 44 participating countries from Europe and North America, in collaboration with the World Health Organisation (Currie *et al.*, 2004; Currie, Samdal, Boyce, & Smith, 2001). Portugal has participated in the survey since 1996, when an initial pilot study adapted to the Portuguese population was carried out, in accordance with the international protocol (www.hbsc.org.com). The study aims to understand further adolescent behaviour in relation to health and to understand health and well-being in the social context, by gathering data that enable national and international comparisons.

The Portuguese study in 2006 included students from grades 6, 8 and 10 within the public education system, with an average age of 14 years *(SD*=1.89). The total sample is 4,877 students from 257 school classes in 125 Portuguese schools selected randomly. In the present study, due to the statistical analisis used, 1,238 students were left out, which accounts for a final total sample of 3,639 adolescents. It is representative of the abovementioned school years and stratified by Administrative Education Regions. Students were distributed in the following manner: 50,4% were girls; 31,7% were in grade 6, 35.,7% in grade 8 and 32,6% in grade 10.

### 2.2 Procedure

The analysis unit used was the class. In each school, classes were randomly selected with the aim to find the needed number in each class, which was proportional to the number provided by the Ministry of Education. Teachers administered the questionnaire in the classroom. Students’ participation was voluntary. Schools and participating classes were selected randomly, out of a list provided by the Ministry for Education. The questionnaires were sent to teachers with the procedure, where it was explicitly asked that the students’ participation was to be anonymous and voluntary. The students that did not wish to complete the questionnaire could leave the classroom.

### 2.3 Measures and Variables

The HBSC Survey 2006 was used for the data gathering, in accordance with the respective protocol. Amongst other, this questionnaire provides demographic data, well-being indicators (life quality related with health, happiness and life satisfaction) and indicators on peer relations (Matos *et al.*, 2006; Currie *et al.*, 2004; Currie, Samdal, Boyce & Smith, 2001). In this study, variables associated with adolescents’ relations with peer groups, their relations with parents, school environment and behaviours of risk, violence and well-being were used.

### 2.4 Statistical Analysis

With the aim to analyse the proposed explanation model, the SEM was used, as a means to assess the quality of mediation of a group of variables. The statistical software EQS, Structural Modelling Software, version 6.1 was used.

Before completely testing the model, it was necessary to test the model partially, through a confirmatory factorial analysis (CFA). Three mediation models were tested: the independent mediation model, which tested the relation of the mediation quality between the independent variables, defined by peer influence, through friends with risk behaviours, made up of five indicators about type of friends, friends with protective behaviours; three indicators about friends’ protective behaviours, communication with friends comprised of three indicators and friendship quality, comprised of ten indicators about friendship quality. The mediating mediation model was comprised of the variables related with adolescents’ relation with parents through communication with the father and the mother, as well as, parental monitoring, comprised of five indicators associated with what parents know about their children. Finally, the dependent mediation model, tested the quality of mediation between the dependent variables, namely low involvement in risk behaviours, comprised of three indicators associated with tobacco, alcohol and drug use; low involvement in violent behaviours, comprised of three indicators associated with provocation, carrying a weapon and involvement in fighting; health, comprised of eleven indicators related with physical and psychological symptoms; well-being, comprised of twelve indicators associated with happiness, life satisfaction and adolescents’ quality of life; and feelings about school, comprised of one indicator (Bentler, 1995).

After the mediation models’ analysis some indicators with saturations lower than. 40 were eliminated. In total eight indicators were eliminated. Covariances between error measures were also introduced, in a total of eight covariances, two in the independent mediation model and six in the mediating mediation model.

## 3. Results

### 3.1 Analysis of the Mediation Model

In the realisation of the model, 1,238 participants were eliminated from the sample. The results obtained in relation to the adequacy of the explanation model proposed showed that it presented lower levels of adequacy (*see* Table 1, step 1). However, the analysis of the results obtained in the Langrange Multiplier test (LM test), a test that assesses the need to add new parameters to the model (Bentler, 1995), showed that the introduction of some connections between factors, would decrease significantly the value of the qui-square, amongst other, the parental monitoring factor, the communication with parents factor and the factor on less involvement in violence behaviours. Amongst the factor on less involvement in risk behaviours and the factor friends with risk behaviours. Amongst the factor low involvement in violence behaviours, the factor on friends with protective behaviours and the factor on less involvement in risk behaviours. Amongst the factor on health and the factor on friendship quality, the communication with friends factor, less involvement in risk behaviours and the factor on well-being, and amongst the factor on feeling about school and less involvement in risk behaviour. A decision was made to add them and to re-evaluate the model (*see* Table 1, step 2). After the introduction of these parameters, the results show better levels of adequacy of the model. Finally, the results obtained in the Wald test, which show the non significant m odel parameters (Bentler, 1995), were analysed. These showed the existence of some non significant relations, which were eliminated, namely between communication with parents and friends with protective behaviours; between parental monitoring, friends with risk behaviours and communication with friends; between lower involvement in risk behaviours and communication with parents; between lower involvement in risk behaviours and parental monitoring; and between health and parental monitoring. The results obtained after the elimination of these parameters are shown in Table 1, step 3.

**Table 1:** Adjustment index

	χ2 (g.l.)^1^	CFI^2^	NNFI^2^	RMSEA (90% I.C.)^2^	SRMR
**Step 1**	358.37 (156)	.87	.86	.036 (.034-.037)	.063
**Step 2**	480.07 (242)	.91	.91	.031 (.029-.032)	.046
**Step 3**	467.27 (237)	.92	.91	.030 (.028-.032)	.045

shows that the procedures undertaken have improved the adjustment index of the structural model. It can be seen that the adjustment index are acceptable and that the model shows a good adequacy.

The standardised solution obtained (Figure 1) allows to confirm that the factors with a stronger impact in relation to lesser involvement in risk behaviours are having friends with risk behaviours (*β*=-.83). The negative indicator shows that the lower the number of friends with risk behaviours, the lower the involvement in risk behaviours. For a lower involvement in violence behaviours, the factors with a greater impact are a lesser involvement in risk behaviours (*β*=.58), followed by friends with protective behaviours (*β*=.35) and communication with parents (*β*=-.21). Following this, the lower the involvement in risk behaviours and the greater number of friends with protective behaviours, the lower the involvement in violence behaviours. On the other hand, the negative Beta figure shows that the worse the communication with parents, the lower the adolescents’ probability in not getting involved in violence behaviours. The factors with a greater impact on health are well-being (*β*=.54), a lower involvement in risk behaviours (*β*=.13) and friendship quality (*β*=-.08). These demonstrate that the greater the well-being, the lower the involvement in risk behaviours and the worse the quality of friendships, the greater the number of health behaviours. The factor with a greater impact on well-being was communication with parents (*β*=.49), which demonstrated that the better the communication with parents, the greater the number of well-being feelings. And in relation to the feeling about school, the lower involvement in risk behaviours is the factor with a greater impact (*β*=.34), meaning that the lower the involvement in risk behaviours, the more adolescents enjoy school.

In relation to mediating factors, for the communication with parents, the factor with a greater impact was communication with friends (*β*=.29) and friends with risk behaviours (*β*=-.18), which suggests that the better the communication with friends and the lower the number of friends with risk behaviours, the better the communication with parents. Communicating easily with parents emerges as a mediating effect between easy communication with friends and involvement in violence behaviours and well-being; and between the type of friends and the violence behaviours and well-being. This relation shows that the easier the communication with friends is, the easier the communication with parents will be and the better adolescents will feel (greater well-being). The type of friends shows a similar effect: the lower the number of friends with risk behaviours, the easier the communication with parents will be and the greater well-being. On the other hand, the relation between the same variables and the violence behaviours is negative, meaning that, the harder the communication with parents, the lower the involvement in risk behaviours. For parental monitoring, the lower involvement in violence behaviours (*β*=.36) and communication with parents (*β*=.33) show that the lower the involvement in violence behaviours and better communication with parents, the more parents tend to monitor adolescents’ behaviours.

Factors’ explained variances, as well as residual ones are presented in Table 2. It shows that having less friends with risk behaviours, explains 67% of the variance of lower involvement in risk behaviours. Being less involved in risk behaviours, worse communication with parents and friends with a protective behaviour explain 45% of the variance of lower involvement in violence behaviours. Feelings of well-being, a lower involvement in risk behaviours and having low quality friendships explain 34% of the variance of health behaviours. A good communication with parents explains 26% of the variance of adolescents’ feelings of well-being. And a lesser involvement in risk behaviours explains 13% of the variance of feelings about school. Concerning the mediating factors, it was found that communication with friends and having less friends with risk behaviours explains approximately 10% of the variance of communication with parents and less involvement in violence behaviours; whilst good communication with parents explains 21% of the parental monitoring variance.

**Table 2:** Explained variance

Factor	R2	Disturbance
**Communicating with parents**	.10	.95
**Parental monitoring**	.21	.89
**Lower involvement in risk behaviours**	.67	.57
**Lower involvement in violence behaviours**	.45	.74
**Health**	.34	.81
**Well-being**	.26	.86
**School**	.13	.93

In relation to the correlation between independent factors, all correlations are positive and significant, with the exception of the correlation between friends with risk behaviours. Friendship quality was not estimated, as it emerged as a non-significant independent variable in the mediation model.

**Table 3:** Correlations

	Friends with risk behaviours	Friends with protective behaviours	Communication with friends	Friendship quality
**Friends with risk behaviours**	___			
**Friends with protective behaviours**	.025*	___		
**Communication with friends**	.12*	.11*	___	
**Friendship quality**	----	.51	.28*	___

* p≤05

## 4. Discussion

The aim of the present study was to confirm the influence of the peer group in risk behaviours, violence, health, well-being and in adolescents’ feelings about school; as well as, whether the relationship with parents may mediate that influence, through an explanatory model undertaken by a structural equation model.

Several studies show the important role played by peers in adolescents’ behaviours (Glaser, Shelton & Bree, 2010; Simões, Matos & Foguet, 2006).

Undoubtedly, friends are one of the most important contexts throughout adolescence. They prevent feelings of loneliness, influence well-being, happiness, health, they help promoting good school achievements and to acquire essential social skills for adult life (Tomé, Matos & Diniz, 2008; Hughes, Dyer, Luo & Kwok, 2009; Camacho, Tomé, Matos, Gamito & Diniz, 2010). On the other hand, friends also emerge as the variable most frequently associated with involvement in risk behaviours (Glaser, Shelton & Bree, 2010; Padilla, Walker & Bean, 2009; Sieving, Perry & Williams, 2000). That influence may be prevented through specific health promotion interventions, which include peers and parents. To prevent that influence presupposes to know what variables are included in that process and which of those may have a mediating role, may influence positively and may diminish negative factors.

The relationship with parents is indicated as a protective variable towards the involvement in risk behaviours and the increase of adolescents’ health and well-being, being it a factor that may also mediate the relationship between adolescents and their peers (Newman, Harrison & Dashiff, 2008; Alvarez, Martin & Vergeles, 2003). Parental communication and monitoring are two faces of that relation, which are mostly identified as enablers of well-being and protective of the involvement in behaviours, which may endanger health. There are a number of studies that show the importance of the positive relation with parents and their protective role, through the proximity between parents and children (Sean, 2010; Luk, Farhat, Ianoti, & Simons-Morton, 2010). That way, the easiness of communication with parents and the fact that parents know of their children’s activities may be the two most important variables, in the mediation of peers’ influence.

In the present study, it was expected to see that parents have a mediating role in the influence by the peer group in relation to adolescents’ behaviours. But the results achieved through the proposed model have not confirmed that role, in relation with to the communication and parental monitoring variables. Communication with parents showed a mediating effect, but the same did not happen for parental monitoring. These effects are evident between communication with parents, the type of friends with risk behaviours and communication with friends, towards less involvement in violence behaviours and well-being. The mediating relations found show that the lower the number of friends with risk behaviour that adolescents have, the easier communicating with parents will be and, in its turn, the higher levels of well-being they will have. The same happens for the mediation with communication with friends. But concerning the relation between the type of friends with risk behaviours and communication with friends, with communication with parents and a lower involvement in risk behaviours, the relation was negative. Consequently, a harder communication with parents, leads to lower levels of involvement in violent behaviours. This negative relation between communicating with parents and a lower involvement in violence behaviours may be associated with the strong impact that the indicators used in the relation between adolescents and the peer group had, in their behaviours; which may have annulled the positive impact of communication with parents in relation to those behaviours. This effect suggests that having an easy communication with friends and having less friends with risk behaviours are protective factors in the involvement in violent behaviours, without needing the mediation of easy communication with parents.

Having a higher number of friends with more risk behaviours also emerges as a factor with a high impact in involvement in risk behaviours, which is in line with several studies that have identified peers as the variable with the greatest influence in the involvement in such behaviours (Glaser, Shelton & Bree, 2010; Sieving, Perry & Williams, 2000). On the other hand, the factor with a greatest impact in low involvement in violence behaviours is a low involvement in risk behaviours and having a higher number of friends involved in protective behaviours. This means that, having friends with little risk behaviours and having friends with protective behaviours, prevent violence and risk behaviours.

A lower involvement in risk behaviours is yet identified as the variable, which positively influences adolescents’ health, together with feelings of well-being; and the variable with influences positively, adolescents’ feelings about school. Taking into account that adolescents seem to choose to be less involved in risk behaviours when they have friends that are not involved in risk behaviours; although peers’ influence is indirectly related, it is very important for adolescents’ health and well-being, as suggested in other studies with similar results (Tomé, Matos & Diniz, 2008; Laftman & Ostberg, 2006; Ueno, 2004).

Parents’ role seems to be more important for the variables associated with adolescents’ well-being and health (Camacho *et al.*, 2010; Newman, Harrison & Dashiff, 2008), considering that communication with parents emerges as the factor with a stronger impact in adolescents’ well-being, which has a stronger impact in health. This presupposes that a better communication with parents means stronger feelings of well-being and, as a result, healthier adolescents.

Parental monitoring does not emerge as a significant variable neither in the mediation of risk and violent behaviours or the promotion of well-being, health or feelings about school. It is reinforced by communication with parents and it is dependent upon a lower involvement in violence behaviours. That dependency shows an unexpected result. Contrary to what is documented in literature (Camacho *et al.*, 2010; Newman, Harrison & Dashiff, 2008), it seems that parental monitoring is greater when adolescents’ involvement in violence behaviours is lower and not the opposite. But we cannot ignore their essential role in parents’ relationship with adolescents (Huebner & Howell, 2003) and take into account the fact that there may be differences associated with gender, age and other variables (Westling *et al.*, 2008), which may influence the results found and limit the impact of the variables analysed.

Another indicator, which did not show an impact in the studied variables, is quality of the friendship, despite the fact that literature shows that the strongest the quality and reciprocity, the more influencing it can be (Glaser, Shelton & Bree, 2010; Mercken *et al.*, 2010). Regardless of this, it shows a positive correlation with communication with friends and in friends with a stronger involvement in protective behaviours, which enhances the friendships’ role in maintaining a positive influence. The negative impact of the quality of the friendship, although it is weak, shows that when the friendship is of low quality, adolescents tend to focus in other areas, such as health.

The results show that the role of peers may be relevant to the risk behaviours, violence, well-being, health and feelings about school, directly and indirectly. Influence, whether positive or negative, is associated with the type of behaviours adopted by friends. As a result, friends that have a higher involvement in risk behaviours have a higher probability in influencing negatively their peers; whilst friends that have more protective behaviours and more easiness in communicating, strengthened by friendships with quality have higher probability of influencing positively their peers. Parents’ role must not be ignored in the mediation of that influence; however it did not emerge as having as strong an impact as expected.

Health promotion interventions targeted at adolescents should take into account the important and positive role that peers may have in the adoption of a healthy lifestyle.

The model presented aimed to understand the influence of peers in adolescents’ behaviours and the role of parents was integrated as a mediation factor, which turned out to be not as significant as expected. For future studies, whose aim is to explore further the relation between these two essential contexts for adolescents’, it is recommended that the relation with peers is transferred to the mediation role and between the relation with parents and their behaviours. Those results could potentially highlight the interaction between both contexts, differently.

## 5. Conclusion

The aim of the present study was to analyse the influence of the peer group in risk behaviours, violence, in health, in well-being and in adolescents’ feelings about school; as well as, whether the relationship with parents may mediate that influence, through an explanatory model undertaken by a structural equation model.

The most important conclusion to be drawn from this study was that peers’ negative or positive influence is associated with peers with risk or protective behaviours, respectively.

Adolescents’ communication with parents has a stronger impact in their health and well-being.

Parental monitoring is influenced by adolescents’ non-involvement in violent behaviours and easiness in communicating with parents.

## 6. Limitations

Some of the limitations found in the present study include the fact that the questionnaire is made up of category-type questions, which make the statistical analyses difficult. The fact that a number of participants were lost throughout the analysis was also a negative factor in obtaining and exploring results. Those participants were excluded because they did not responde to the variables used in the study. This type of transversal study does not enable us to find cause effects, only to verify the association between the variables.

## References

[ref1] Ackard D. M, Neumark-Sztainer D, Story M (2006). Parent – child connectedness and behavioral and emotional health among adolescents. American Journal of Preventive Medicine.

[ref2] Alvarez J. M, Martins A. F, Vergeles M. R (2003). Substance use in adolescence: Importance of parental warmth and supervision. Psicothema.

[ref3] Ary D. V, Duncan T. E, Biglan A (1999). Development of adolescente problema behavior. Journal of Abnormal Child Psychology.

[ref4] Baptista I, Tomé G, Matos M. G (2008). A Escola. Jovens com Saúde - Diálogo com uma geração.

[ref5] Bentler P. M (1995). EQS Structural Equations Program Manual.

[ref6] Bongers I. L, Koot H. M, Van der Ende J (2008). Predicting young adult social functioning from developmental trajectories of externalizing behaviour. Psychological Medicine.

[ref7] Camacho I, Tomé G, Matos M (2010). A escola e os adolescentes: Qual a influência da família e dos amigos?. Revista de Psicologia da Criança e do Adolescente.

[ref8] Currie C, Samdal O, Boyce W (2001). HBSC, and WHO cross national study: research protocol for the 2001/2002 survey.

[ref9] Currie C, Roberts C, Morgan A (2004). HBSC, and WHO cross national study: research protocol for the 2001/2002 survey.

[ref10] Demir M, Urberg K. A (2006). Friendship and adjustment among adolescents. Journal of Experimental Child Psychology.

[ref11] Gaspar T, Matos M (2008). Qualidade de vida em crianças e adolescentes versão portuguesa dos intrumentos Kidscreen, 52.

[ref12] Glaser B, Shelton H. K, Bree M (2010). The Moderating Role of Close Friends in the Relationship between Conduct Problems and Adolescent Substance use. Journal of Adolescent Health.

[ref13] Huebner A, Howell L (2003). Examining the relationship between adolescent sexual risk-taking and perceptions of monitoring, communication, and parenting styles. Journal of Adolescent Health.

[ref14] Hughes J. N, Dyer N, Luo W (2009). Effects of peer academic reputation on achievement in academically at-risk elementary students. Journal of Applied Developmental Psychology.

[ref15] Kuntsche E, Knibbe R, Gmel G (2005). Why do young people drink? A review of drinking motives. Clinical Psychology Review.

[ref16] Laftman S. B, Östberg V (2006). The pros and cons of social relations: An analysis of adolescents’ health complaints. Social Science & Medicine.

[ref17] Larsen H, Engels R. C, Souren P. M (2010). Peer influence in a micro-perspective: Imitation of alcoholic and non-alcoholic beverages. Addictive Behaviors.

[ref18] Li X, Stanton B, Feigelman S (2000). Impact of perceived parental monitoring on adolescent risk behavior over 4 years. Journal of Adolescent Health.

[ref19] Luk W. J, Farhat T, Iannotti J. R (2010). Parent – child communication and substance use among adolescents: Do father and mother communication play a different role for sons and daughters?. Addictive behaviors.

[ref20] Matos M (2006). Equipa do Aventura Social. A saúde dos adolescentes Portugueses – Hoje e em 8 anos – Relatório Preliminar do estudo HBSC 2006.

[ref21] Mercken L, Snijders T, Steglich C (2010). Dynamics of adolescent friendship networks and smoking behavior. Social Networks.

[ref22] Mulye T, Park M, Neson C (2009). Trends in Adolescent and Young Adult Health in the United States. Jounal of Adolescent Health.

[ref23] Newman K, Harrison L, Dashiff C (2008). Relationships between parenting styles and risk behaviors in adolescent health: An integrative literature review. Revista Latino-Americana de Enfermagem.

[ref24] Padilla-Walker L. M, Bean R. A (2009). Negative and positive peer influence: Relations to positive and negative behaviors of African American, European American, and Hispanic adolescents. Journal of Adolescence.

[ref25] Parker J. S, Benson M. J (2004). Parent-adolescent relations and adolescent functioning: Self-esteem, substance abuse, and delinquency. Adolescence.

[ref26] Petraitis J, Flay B. R, Miller T. Q (1995). Reviewing theories of adolescent substance use: Organizing pieces in the puzzle. SPsychological Bulletin.

[ref27] Piko B, Hamvai C (2010). Parent, school and peer-related correlates of adolescents’ life satisfaction. Children and Youth Services Review.

[ref28] Rai A, Stanton B, Wu Y (2003). Relative Influences of Perceived Parental Monitoring and Perceived Peer Involvement on Adolescent Risk Behaviors: An Analysis of Six Cross-sectional Data Sets. Journal of Adolescent Health.

[ref29] Ruini C, Ottolini F, Tomba E (2009). School intervention for promoting psychological well-being in adolescence. Journal of Behavior Therapy and Experimental Psychiatry.

[ref30] Sen B (2010). The relationships between frequency of family dinner and adolescent problem behaviors after adjusting for other family characteristics. Journal of Adolescence.

[ref31] Sieving E. R, Perry L. C, Williams L. C (2000). Do friendships changes behaviors, or Do behaviors change friendships? Examining paths of influence in young adolescents´alcohol use. Journal of Adolescent Health.

[ref32] Simões C, Matos M, Foguet J (2006). Consumo de Substâncias na Adolescência: Um Modelo Explicativo. Psicologia, Saúde & Doenças.

[ref33] Simons-Morton B, Chen R (2005). Latent growth curve analyses of parent influences on drinking progression among early adolescents. Journal of Studies on Alcohol.

[ref34] Sumter S, Bokhorst C, Steinberg L (2009). The developmental pattern of resistance to peer influence in adolescence: Will the teenager ever be able to resist?. Journal of Adolescence.

[ref35] Tobler L. A, Komro A. K (2010). Trajectories or Parental Monitoring and Communication and Effects on Drug Use among Urban Young Adolescents. Journal of Adolescents Health.

[ref36] Tomé G, Matos M, Dinis A, M Matos (2008). Consumo de substâncias e felicidade nos adolescentes. Consumo de Substâncias: Estilo de Vida? ÀProcura de um estilo?.

[ref37] Ueno K (2004). The effects of friendship networks on adolescent depressive symptoms. Social Science Research.

[ref38] Vaquera E, Kao G (2008). Do you like me as much as I like you? Friendships reciprocity and its effects on school outcomes among adolescents. Social Science Research.

[ref39] Westling E, Andrews J, Hampson S (2008). Pubertal Timing and Substance Use: The Effects of Gender, Parental Monitoring and Deviant Peers. Journal of Adolescent Health.

